# Complex(iti)es of the ubiquitous RNA-binding CSP41 proteins

**DOI:** 10.3389/fpls.2014.00255

**Published:** 2014-06-06

**Authors:** Dario Leister

**Affiliations:** ^1^Department Biology I, Plant Molecular Biology (Botany), Ludwig-Maximilians-University MunichMartinsried, Germany; ^2^Department of Plant and Environmental Sciences, Copenhagen Plant Science Centre, University of CopenhagenCopenhagen, Denmark

**Keywords:** Arabidopsis, chloroplast, gene expression, RNA, RNA-binding protein, transcription, translation

Photosynthetic eukaryotes encode two copies of the CSP41 (Chloroplast Stemloop binding Protein of 41 kDa) protein that are of cyanobacterial origin. In Arabidopsis thaliana, the two CSP41 proteins belong to the group of most-abundant chloroplast proteins. Multiple functions have been described for CSP41 proteins, including roles in chloroplast rRNA metabolism and transcription. CSP41a and CSP41b interact physically. Recent data show that CSP41b is an essential and major component of high-molecular weight complexes that form in the dark, disassemble in the light, and bind chloroplast mRNAs coding for photosynthetic proteins and some ribosomal RNAs, but not the plastid-encoded RNA polymerase (PEP). This, together with the effects seen in leaves of plants lacking CSP41b, implies that complexes containing CSP41 proteins stabilize untranslated mRNAs and precursor rRNAs. This occurs in a redox-dependent manner and seems to be important in the absence light when the translation is less active. In this scenario, translation and transcription is secondarily affected by the decreased transcript stability.

## CSP41 proteins are abundant and constituents of complexes

CSP41 proteins are highly abundant chloroplast proteins. Zybailov et al. (2008) grouped chloroplast stromal proteins into seven abundance classes and CSP41b is found in the group of highest abundance together with the Calvin cycle enzymes for instance. CSP41b is more abundant than CSP41a, which is found in the group of second highest abundance together with most chloroplast ribosomal proteins. Therefore, it does not come as surprise that CSP41 proteins have been detected in several stromal complexes, but not all of these associations are necessarily of physiological significance in the light of the ubiquity of these proteins.

The first report on CSP41a function described its binding *in vitro* to the 3′ end of the *petD* mRNA (Yang et al., 1995, 1996). Subsequently, CSP41 proteins were also found in preparations that were enriched for the plastid-encoded RNA PEP (Pfannschmidt et al., 2000) or plastid ribosomes (Yamaguchi et al., 2003). However, later studies could not confirm that CSP41 proteins are part of the PEP complex (Suzuki et al., 2004; Pfalz et al., 2006). A ribosome association of CSP41a and b was also observed by Peltier et al. (2006) in their analysis of the stromal proteome in its oligomeric state extracted from highly purified chloroplasts of *Arabidopsis thaliana*. The two CSP41 proteins were each found at three different locations of the stromal colorless native (CN)-PAGE native gels: (i) in a complex larger than 950 kDa most likely associated with 70 S ribosomes, (ii) at 224 kDa, and (iii) at 106–126 kDa. At 224 kDa, the only obvious potential partners are the ribosomal proteins L5 and L31. At 106–126 kDa, CSP41a and b possibly form a heterotrimer. A further proteomics analysis found CSP41b mostly in 0.8–2 MDa fractions of stromal high-molecular-weight (HMW) complexes, together with other proteins like subunits of the 30S part of the plastid ribosome and subunit E2 of the plastid pyruvate decarboxylase (LTA2) (Olinares et al., 2010).

Recently, Qi et al. (2012) found by co-immunoprecipitation experiments that the major interactor of CSP41b is the CSP41a protein. The majority of both CSP41 proteins comigrates in several distinct spots during 2D BN/SDS PAGE, implying that they are present in multimeric protein complexes mainly comprised of these two subunits. Because the HMW CSP41 complexes are disrupted by treatment with RNase, they should be associated with RNAs (Qi et al., 2012). RIP-chip analysis points to chloroplast mRNAs coding for photosynthetic proteins and some ribosomal RNAs, but no tRNAs or mRNAs for ribosomal proteins, as putative ligands of CSP41 complexes (see below). The only other protein, besides CSP41a, found in immunoprecipitates of tagged CSP41b was LTA2 (Qi et al., 2012), corroborating the results of the proteomic study of Olinares et al. (2010). Interestingly, LTA2 is not a highly abundant stromal protein (Zybailov et al., 2008); therefore, this interaction might be specific and not due to contamination.

Taken together, because of their high abundance, CSP41 proteins can be found as contaminants in preparations of several stromal complexes. However, the results obtained by Qi et al. (2012) imply that CSP41 does not functionally interact with PEP or the plastid ribosome, as proposed before. Indeed, CSP41 complexes appear to contain chloroplast mRNAs coding for photosynthetic proteins and some ribosomal RNAs.

## CSP41 functions at the biochemical level

Multiple functions have been assigned to CSP41 proteins. (1) RNase activity, with a preference for 3′ stem-loops (Yang and Stern, 1997; Bollenbach and Stern, 2003a,b). (2) Ribosomal biogenesis, because of the association of CSP41b with pre-ribosomal particles (Beligni and Mayfield, 2008). (3) Plastid transcription (Bollenbach et al., 2009). (4) Cytosolic functions based on its interaction with heteroglycans in the cytosol (Fettke et al., 2011).

Not all of the four tentative functions of CSP41 proteins described above might be physiologically relevant and rather represent artifacts similar to the multiple presences of CP41 proteins in HMW complexes. Moreover, the high abundance of CSP41 proteins argues against a specific catalytic function but points in the direction of a more general function, requiring relative large quantities of the protein. Recently, Qi et al. (2012) demonstrated by RIP-chip analysis that CSP41 can bind various chloroplast RNAs. This includes transcripts for the large Rubisco subunit (*rbcL*), PSI (*psaA, psaB*), and PSII (*psbA, psbC, psbD*) core proteins, and 16S and 23S rRNAs. Therefore, Qi et al. (2012) concluded that the CSP41 proteins might serve to stabilize RNAs. Indeed, in their *in-organello* assay the stability of two tentative target RNAs (one of them 23S rRNA) was found to be decreased in mutants lacking CSP41b. Consequently, such destabilization of the precursors of 23S and 16S rRNA might result in fewer functional ribosomes, and in turn in a decrease of the rate of chloroplast translation. As a further consequence of a reduced translation rate, a decline of the levels of PEP synthesis can be expected and, in turn, of the rate of transcription. Nevertheless, a direct effect on transcription/translation through binding of CSP41 to target transcripts cannot be entirely ruled out yet.

The findings that CSP41 displays endonuclease activity *in vitro* (Yang et al., 1996; Yang and Stern, 1997; Bollenbach and Stern, 2003a,b) and CSP41 proteins stabilize target RNAs *in vivo* are not necessarily mutually exclusive, because the endoribonuclease activity of CSP41 could be highly regulated *in vivo*, e.g., by phosphorylation (Qi et al., 2012). Thus, complexes of CSP41 in its inactive state (without endonuclease activity) might stabilize RNAs by binding to protect them against degradation. Certain conditions could then activate the ribonucleolytic activity of CSP41, leading to the degradation of the target transcripts; however, it remains to be clarified whether CSP41 actually plays a role as RNase *in vivo*. In this context it is interesting to note that changes in the pIs of CSP41b species between dark and light conditions suggests that redox-dependent post-translational modifications of CSP41 might regulate the capacity of CSP41 complexes to bind RNA (Qi et al., 2012).

Immunoprecipitates of tagged CSP41b contain also LTA2, the E2 subunit of the plastid pyruvate decarboxylase (Qi et al., 2012). Interestingly, the counterpart of LTA2 in the green alga *Chlamydomonas reinhardtii*, DLA2, binds *psbA* mRNA and has been implicated in the reciprocal regulation of protein synthesis and carbon metabolism for thylakoid membrane biogenesis (Bohne et al., 2013). Therefore, their *psbA* transcript binding activity might bring together CSP41 proteins and LTA2 in the same complex.

## Mutant analyses of CSP41 functions in plants

The *csp41b* mutation affects the morphology of chloroplasts, photosynthesis and circadian rhythms (Hassidim et al., 2007). Based on the observation that *A. thaliana* mutants without both CSP41 proteins are not viable, Beligni and Mayfield (2008) proposed that CSP41a and CSP41b have redundant functions. However, recent data by Qi et al. (2012) argue in favor of the notion that CSP41a and CSP41b do not have entirely redundant functions and that CSP41b is functionally more important than CSP41a. (1) While loss of CSP41a does not result in obvious phenotypic effects, chloroplast RNA levels and plant performance are impaired when CSP41b is inactive. Moreover, the *csp41ab* double mutant behaves like *csp41b* mutant plants (Qi et al., 2012). (2) Although CSP41 protein complexes seem to contain both proteins in wild-type plants, only CSP41b is essential for their formation. (3) Phylogenetic analysis of CSP41 sequences from *A. thaliana, C. reinhardtii* and the cyanobacterium *Synechocystis* suggest that CSP41a might be less constrained evolutionarily (Qi et al., 2012).

The major CSP41 protein, CSP41b, accumulates predominantly in mature leaves (Fettke et al., 2011). Accordingly, the function of the CSP41 proteins appears to be particularly required in mature leaves, as determined by measurements of translation rate and photosynthetic activity (Qi et al., 2012). Interestingly, mutants with reduced PEP levels show on opposite behavior compared to *csp41b* mutant plants with normal mature leaves but compromised younger leaves (Chi et al., 2008; Chateigner-Boutin et al., 2011). Moreover, the sets of mRNAs that are bound by CSP41 complexes or which are transcribed by the PEP overlap. Therefore, it can be concluded that in young leaves sufficient transcripts are synthesized by PEP such that the function of CSP41 complexes is not required. In older leaves, however, chloroplast gene expression can only be maintained by transcript stabilization through CSP41 complexes. In line with this, it has been described that the stability of chloroplast transcripts increases with the leaf age (Klaff and Gruissem, 1991). In fact, the post-translational modification of CSP41 proteins could represent a development-dependent regulatory mechanism by which the function of CSP41 is controlled.

**Figure 1:**
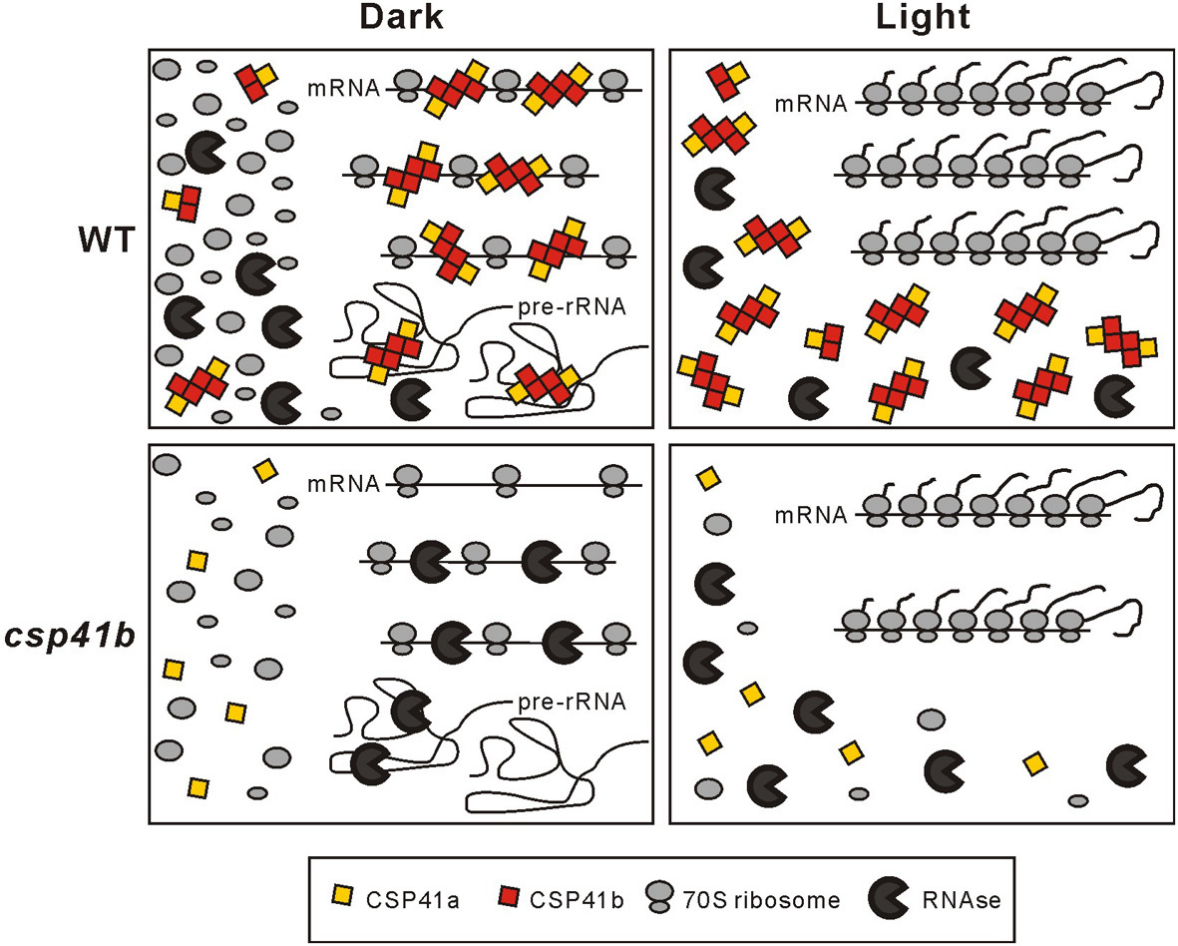
**Model for action of CSP41 protein complexes**. In the dark, CSP41 protein complexes associate with various mRNAs and some pre-rRNAs and protect them from nucleolytic cleavage. Untranslated RNAs not stabilized by CSP41 protein complexes are degraded by ribonucleases. Newly synthesized precursors of rRNAs are rapidly incorporated in the light into functional ribosomes, which in turn stabilize plastid transcripts during translation. In the absence of CSP41b, protection of mRNAs and pre-rRNAs in the dark is impaired and HMW RNA-CSP41 complexes are not formed. In consequence less functional ribosomes and mRNAs are available in the light. Therefore, less photosynthetic subunits are synthesized and the translational capacity is generally decreased. The latter can explain the pleiotropic effects seen in *csp41b* plants. Modified from Qi et al. (2012).

In the light with highest activity of the chloroplast translational machinery the CSP41 proteins fail to form significant amounts of HMW complexes. On the contrary, darkness induces formation of HMW CSP41 complexes that are sensitive to treatment with RNase (Qi et al., 2012). This, together with the assumption that increased polysomal association of transcripts serves to stabilize them in the light (Qi et al., 2012), is the basis for our working model for the mechanism by which CSP41 complexes stabilize mRNAs in the dark (Figure 1). In this model, CSP41 proteins bind in the dark to non-translated mRNAs and rRNA precursors (which are not incorporated in ribosomes) to protect them against degradation. As soon as translation is activated in the light, this would allow the rapid initiation of translation and elongation from these stabilized transcripts, as well as *de-novo* assembly of ribosomes. In the mutants that lack CSP41 complexes, un-translated target mRNAs and precursors of rRNAs are prone to increased degradation. A role of the chloroplast redox state in the regulation of the association of CSP41 proteins with their RNA targets in the dark (and their light-induced dissociation) became evident when studying a photosynthetic mutant line with a more oxidized redox state of the stroma (Qi et al., 2012): here, HMW CSP41complexes can persist also in the light.

## Conclusions

The key to understand the multiple functions of CSP41 proteins probably lies in their abundance. Actually, CSP41 proteins are the most abundant RNA-binding proteins in the chloroplast stroma. Their function becomes critical in mature leaves when transcripts produced by PEP might become limiting and need to be stabilized and protected. Therefore, lack of CSP41 proteins decreases transcripts for photosynthetic proteins and of some ribosomal RNAs, which in turn, appears to result in pleiotropic effects due to a decrease in the translational activity of chloroplasts.

### Conflict of interest statement

The author declares that the research was conducted in the absence of any commercial or financial relationships that could be construed as a potential conflict of interest.
